# Gender-Specific Behaviour in Obesity Stages I-II: Imbalance of Aminothiol Status and Adipomyokine Profile in Subjects with Different Insulin Resistance Severity

**DOI:** 10.1155/2021/9713582

**Published:** 2021-11-24

**Authors:** Jonica Campolo, Ettore Corradi, Marina Parolini, Maria Luisa Di Guglielmo, Alice Rizzardi, Cinzia Dellanoce, Patrizia Tarlarini, Marina Cattaneo, Elena Scioscioli, Maria Giovanna Trivella, Renata De Maria

**Affiliations:** ^1^CNR Institute of Clinical Physiology, ASST Grande Ospedale Metropolitano Niguarda, Milan 20162, Italy; ^2^Clinical Nutritional Unit, ASST Grande Ospedale Metropolitano Niguarda, Milan 20162, Italy

## Abstract

The hyperproduction of oxidative stress and inflammatory biomarkers, which is paralleled by decreased levels of antioxidant and anti-inflammatory mediators, is part of cellular mechanisms that contribute to the disruption of metabolic homeostasis in obesity. Whether gender-specific alterations and gender-restricted associations in these biomarkers underlie the increased cardiometabolic risk in men compared to women is unclear. We enrolled 31 women and 29 men, aged ≥50 and ≤70 years and with body mass index ≥ 30 and <40 kg/m^2^. We assessed the concentrations of aminothiols (cysteine, homocysteine, and glutathione), expression of oxidant/antioxidant balance, adipomyokines (leptin, adiponectin, myostatin, and interleukin-6), markers of chronic inflammation, and vitamin D, an index of nutritional state, in plasma and serum samples by using HPLC, ELISA, and chemiluminescent immunoassay methods. We measured insulin resistance (IR) by the homeostasis model assessment (HOMA) index. Despite comparable levels of visceral adiposity, IR, and a similar dietary regimen, men showed, with respect to women, higher oxidant concentrations and lower antioxidant levels, which paralleled IR severity. Myostatin levels correlated with prooxidant aminothiols among men only. Gender-specific alterations in aminothiol status and adipomyokine profile and the gender-restricted association between these biomarkers and metabolic derangement are consistent with an increased cardiometabolic risk in men compared to age-matched women with stage I-II obesity. Strict control of redox and inflammatory status, even addressing gender-specific nutritional targets, may be useful to prevent obesity-related metabolic alterations and comorbidities.

## 1. Introduction

Obesity is a serious health problem with a worldwide impact on the risk and prognosis of several chronic diseases, such as type 2 diabetes, coronary heart disease, nonalcoholic fatty liver disease, and cancer [1]. Adverse changes associated with weight gain such as visceral obesity and physical inactivity may affect adipose and muscle tissues causing low-grade chronic inflammation and insulin resistance [2].

The mechanisms by which adipose depots expand in response to an excessive caloric intake represent a crucial determinant of the risk of metabolic dysfunction. This expansion is mediated by an increase in adipocyte numbers and/or enlargement of their size, by infiltration of immune cells, such as macrophages [3], into the adipose tissue, and by dysregulation of extracellular matrix remodelling in fat tissue [4]. Sustained obesity as well as chronic inflammation determines abnormal secretion of adipokines and myokines, excessive lipid storage, local hypoxia, and fibrosis [5].

Hyperproduction of proatherogenic, proinflammatory, and prodiabetic adipocytokines, which is accompanied by a decreased production of the anti-inflammatory adiponectin, is part of cellular mechanisms that contribute to the disruption of metabolic homeostasis.

The synthesis of proinflammatory mediators stimulates adipocytes and also other tissues, to generate reactive oxygen species (ROS) through the activation of the NADPH oxidase, increasing in this way oxidative stress [6–8].

Excess ROS attenuates vascular nitric oxide bioavailability and leads to endothelial dysfunction, facilitates oxidation of low-density lipoprotein involved in atherosclerotic plaque formation, suppresses the activity of several important transcription factors of insulin gene, and decreases insulin response [9].

Susceptibility to oxidative damage is even greater in obese subjects because their antioxidant defence is depleted; the activity of antioxidant enzymes including superoxide dismutase, glutathione peroxidase, and catalase was decreased in obese individuals compared to normal weight subjects [7, 10], and a prooxidant/antioxidant burden, shifted towards oxidation, was found in morbidly obese patients compared to lean healthy controls [11, 12].

Although the oxidative unbalance in obesity is well described, it is not clear if gender may affect redox homeostasis and inflammation in those people at high risk of metabolic complications.

We aimed to explore, in a series of middle-aged nondiabetic subjects with stage I and II obesity, differences in systemic redox balance, nutritional status, and secretion of adipomyokines with respect to gender and severity of insulin resistance, a cardinal risk factor for the development of cardiometabolic disease.

## 2. Materials and Methods

### 2.1. Study Population

Among 218 subjects screened at the Clinical Nutritional Unit of ASST Grande Ospedale Metropolitano Niguarda, 60 (31 females, 29 males) had no exclusion criteria and consented to participate in the study.

We excluded patients with obesity aged <50 and >70 years, with a BMI ≥ 40 kg/m^2^, with kidney (estimated glomerular filtration rate < 15 mL/min/1.73 m^2^) or liver (AST-ALT × 2 times upper normalcy range) dysfunction, and with active cancer (diagnosed <5 years before enrolment), overt diabetes (fasting glucose ≥ 126 mg/dL or glycated haemoglobin ≥ 48 mmol/mol), and history of inflammatory or autoimmune diseases. In the three months preceding the study, patients had not taken any antioxidant supplements, anti-inflammatory drugs, glucocorticosteroids, or antibiotics. All women were postmenopausal and were not receiving hormonal replacement therapy. Five of them were taking vitamin D.

The enrolled population had a median age of 59 [56-63] years and a BMI of 33 [13–18]. Thirty-two patients (53%) had hypertension (systolic and diastolic blood pressure ≥ 140 and 90 mmHg, respectively, or on antihypertensive medications), 22 (37%) had dyslipidemia (LDL ≥ 160 mg/dL or current treatment with lipid-lowering medications), and 9 (15%) were smokers.

Subjects were on a maintenance weight loss program through lifestyle advice management combined with a dietary regimen based on the principles of the Modern Mediterranean Diet Pyramid [19].

The study protocol conformed to the principles of the Helsinki Declaration and was approved by the Local Ethics Committee.

### 2.2. Anthropometric Measurements

Height was measured with a professional stadiometer (sensitive to 0.1 cm difference) in the upright position with bare feet; weight was measured on an electronic scale standing upright and stationary without shoes and with light underwear. Body mass index (BMI) was calculated as body weight (kg)/height (meters) squared.

The visceral adiposity index (VAI), a gender-specific empirical-mathematical model, was calculated using anthropometric (BMI and waist circumference) and functional (triglycerides and HDL cholesterol) parameters indicative of fat distribution and function [20].

### 2.3. Blood Sample Processing

Eligible subjects attended our Institute in the fasting state for peripheral blood sampling. Venous blood was collected in EDTA and in serum separator tubes. Blood in EDTA (3 mL) was immediately centrifugated at 4000 rpm, 4°C for 15 minutes in order to obtain plasma aliquots, while blood collected in serum separator tubes (3 mL) was kept at room temperature for 30 minutes to allow sample coagulation and then centrifugated at 4000 rpm for 15 minutes.

### 2.4. Biochemical Analyses

Fasting glucose and insulin were measured by standard laboratory procedures, in order to assess the Homeostatic Model Assessment (HOMA) index and quantify insulin resistance (IR). The HOMA score was calculated as (fasting glucose (mg/dl) × fasting insulin (*μ*U/mL))/405. Based on a cut-off obtained in a nondiabetic Mediterranean population [21], a HOMA ≥ 3.8 identified subjects with IR.

Vitamin D (25-hydroxyvitamin D3), a marker of nutritional status, was measured in serum by a chemiluminescent immunoassay (LIAISON® 25 OH Vitamin D Total Assay, DiaSorin).

The aminothiol redox status, index of oxidant and antioxidant balance, was assessed in plasma as total (t-) and reduced (r-) forms of cysteine (Cys), homocysteine (Hcy), and glutathione (GSH). The total forms include free, disulfide, and protein-bound aminothiols while the reduced forms only the free component. We measured total aminothiol concentrations by an isocratic high-performance liquid chromatographic (HPLC) method validated in our laboratory [22] that uses ammonium-7-fluorobenzo-2-oxa-1,3-diazole-4-sulphonate as a derivatizing agent. Plasma reduced forms were instead assessed by mixing plasma samples with 10% trichloroacetic acid (1 : 1, *v*/*v*), containing EDTA 1 mM, before derivatizing step. The aminothiol separation was performed on a 5 *μ*m Discovery C-18 analytical column (250 × 4.6 mm I.D.), eluted with a solution of 0.1 mol/L potassium dihydrogenphosphate-acetonitrile (92 : 8, *v*/*v*), pH 2.1, at a flow rate of 1 mL/min. Fluorescence intensities were measured at *λ*ex = 355 nm and *λ*em = 450 nm with a JASCO FP-4025 detector. The oxidized forms (-ox) of all aminothiols were calculated as the difference between t- and r- forms and were the expression of disulfides and protein-bound oxidized aminothiols.

Adipomyokines, markers of inflammatory profile, were measured in serum samples by an antigenic immunoassay procedure based on enzyme-linked immunosorbent assays: leptin (EIA-2395, DRG Instruments, GmbH), adiponectin (EIA-4177, DRG Instruments, GmbH), myostatin (SEB653Hu, Cloud-Clone Corp.), and interleukin-6 (D6050, R&D Systems, Inc.). Analyses of adipomyokines were carried out in a microplate reader Tecan Infinite F200.

### 2.5. Statistical Analysis

Enrolment was based on the estimated proportion of subjects presenting a HOMA-IR ≥ 3.8. Assuming that 30% of the subjects have the factor of interest, the study would require a sample size of 60 for estimating the expected proportion with 12% absolute precision in 95% CI, with alpha = 0.05.

Data are expressed as the median and interquartile range (I-III) for continuous variables or as number and frequency percent for categorical variables. Normality assumptions of variable distributions were examined by Shapiro-Wilk's test. Between-group differences were tested by unpaired Student's *t*-test for continuous variables, or by Mann–Whitney test for skewed data, and by the chi-squared test or Fisher exact test for categorical variables.

The relationships between adipomyokines and other clinical and biochemical parameters were tested using Pearson's correlation test or Spearman's rank correlation test, as appropriate.

We used univariable and multivariable logistic regression analyses to explore risk factors associated with gender (men vs. women) and IR (HOMA ≥ 3.8 vs. HOMA < 3.8); all variables with a *P* value ≤ 0.1 in the univariable analysis were included in the multivariable model. The results from logistic regression models were reported as odds ratios (OR) with the corresponding 95% confidence intervals (CI).

A two-tailed *P* value < 0.05 was considered significant. Statistical analyses were performed using SPSS ver. 24.0 software package (IBM SPSS, New York, USA).

## 3. Results

### 3.1. Gender Differences in Aminothiol/Adipomyokine Profiles

Table 1 reports the anthropometric and clinical characteristics of the enrolled population categorized by gender. Female and male groups were comparable in age, BMI, VAI, HOMA index, glucose, glycated haemoglobin, insulin, AST, ALT, LDL cholesterol, and triglycerides but not in HDL cholesterol concentrations that, as expected, were increased in women compared to men. No between-gender differences were found in the prevalence of hypertension, dyslipidemia and smoking habit or drugs, and vitamin D supplementation.

**Figure 1:**
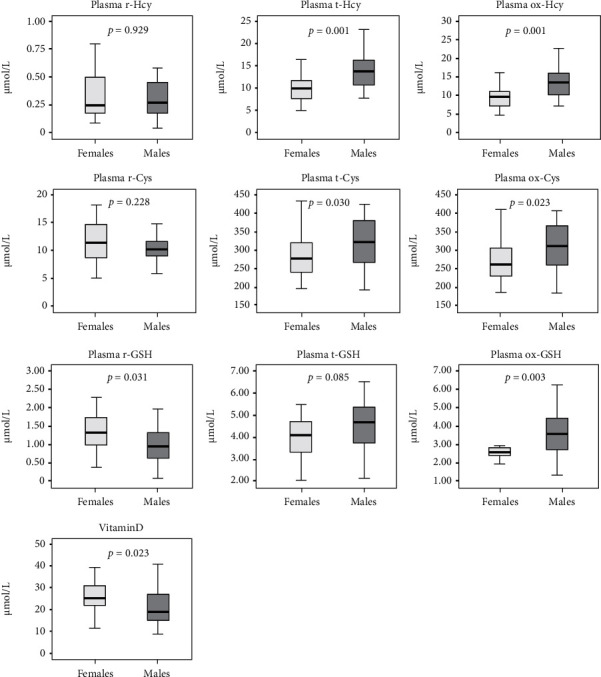
Differences in aminothiol contents and vitamin D concentrations between females and males. Cys: cysteine; Hcy: homocysteine; GSH: glutathione; t-: total; r-: reduced; ox-: oxidized.

**Figure 2:**
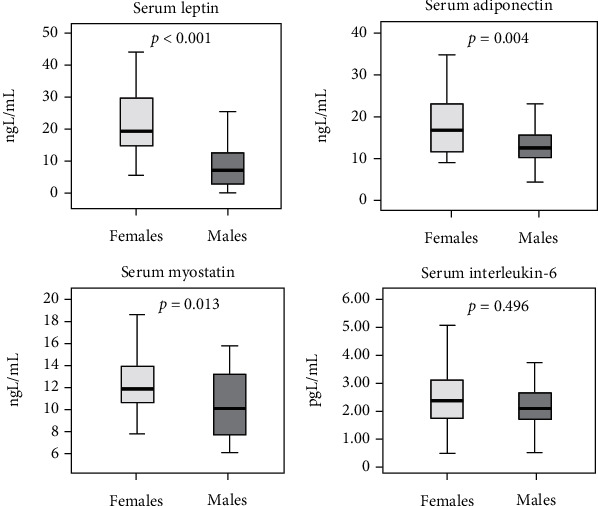
Differences in adipomyokine levels between females and males.

The total forms of Hcy and Cys and ox-Hcy, ox-Cys, and ox-GSH were significantly higher in males than in females; conversely, r-GSH and vitamin D concentrations were lower in men with respect to women (Figure 1), even after exclusion of the 5 females supplemented with vitamin D. Leptin, adiponectin, and myostatin levels were instead higher in females than in males while serum IL-6 did not differ between groups (Figure 2).

The correlations by gender between aminothiol status and the other biochemical parameters under study are presented in Table 2. In the male group (A) only, myostatin was positively correlated with t-Hcy, ox-Hcy, r-Cys, t-GSH, and ox-GSH levels. No other relations were observed either in men or in women.

### 3.2. Association of Aminothiols/Adipomyokines with Gender

The univariable logistic regression analysis for the association between male gender and aminothiol/adipomyokine parameters is depicted in Table 3. High levels of t-Hcy, t-Cys, ox-Hcy, ox-Cys, and ox-GSH and low concentrations of leptin, adiponectin, myostatin, and vitamin D were associated with the male group. ox-Hcy and ox-Cys, due to high collinearity with their total forms, were not evaluated in the multivariate analysis. By multivariable logistic regression, only ox-GSH, leptin, adiponectin, and myostatin were independently related to male gender.

### 3.3. Gender-Related Associations of Aminothiols/Adipomyokines with Insulin Resistance

Based on HOMA values, we categorized our population into two groups: subjects without IR (HOMA < 3.8) and those with severe IR (HOMA ≥ 3.8).

We tested the relationship of biochemical parameters to severe IR by logistic regression analysis (Table 4). The univariable test showed that high t-Cys, ox-Cys, leptin, and IL-6 levels and lower adiponectin and vitamin D concentrations were associated with severe IR. The multivariable analysis, adjusted also for gender (Table 5), indicates that t-Cys, leptin, and IL-6 concentrations remained the only variables related to HOMA index ≥ 3.8.

## 4. Discussion

In subjects with stage I-II obesity despite similar age, visceral adiposity, HOMA index, and dietary regimen, we found gender-related differences in aminothiol and adipomyokine concentrations. These alterations, indicative of an unfavourable oxidative stress and inflammatory profile, were associated with IR severity in the male group only.

In our series, men had higher t-Hcy, ox-Hcy, t-Cys, ox-Cys, and ox-GSH and lower r-GSH levels with respect to age-matched, postmenopausal women. Moreover, men had also lower leptin, adiponectin, and myostatin concentrations than women.

Hcy and Cys are prooxidant mediators able to produce free radicals through autoxidation [23] or by stimulation of NADPH-oxidase and inducible nitric oxide synthase [24], while r-GSH is the most important antioxidant marker in our organism. The increment of the total and oxidized aminothiol forms and the depletion of r-GSH suggest the presence of higher oxidative stress in men than in women in this middle-aged population.

Gender differences in oxidative stress markers and antioxidant enzymes are reported by different authors, but the results are few and controversial. Some authors reported that women with metabolic syndrome have a better antioxidant status, expressed as total antioxidant capacity, and higher apolipoprotein A levels compared with men, suggesting indirectly the existence of a higher oxidative stress index in men with metabolic syndrome [25]. Brunelli et al. found a significant between-gender difference in oxidative stress markers in healthy volunteers: production of free radicals was higher in women than in men, independently of woman hormonal status [26]. In young healthy adults, Montes-Nieto et al. showed that plasma TBARS concentrations, indices of lipid hydroperoxide, were different in obese versus nonobese male subjects, but not between men and women of each group [27].

A recent work by Choromańska et al. [11] suggested an age and gender effect on redox homeostasis in obese patients and lean controls. In particular, oxidative stress increased with age and was more intense in men than in women, at the same age, in both groups of subjects.

Our women appear protected from oxidative insult with respect to men, even if their estrogen activity is reduced. It is known that after menopause, the accumulation and distribution of fat in women shift towards abdominal obesity like men and that this change makes the risk of metabolic complications similar in both gender [28, 29]. Although visceral adiposity, as expressed by VAI, overlapped between genders, in our population, we found an association between increased levels of Cys, leptin, and IL-6 and severe IR only in men. Cys is produced mainly by endogenous synthesis from its precursor methionine, by dietary intake [30] and, in a lesser amount, by protein turnover. High plasma t-Cys levels correlate strongly with obesity [31] and are linked to obesity-related disorders, such as cardiovascular diseases [32].

Therefore, a personalized gender-specific diet with methionine restriction could be a good nutritional strategy to modulate plasma biomarker levels in subjects with obesity and to prevent the development of IR and related disorders.

We confirmed that the well-described sex dimorphism in leptin and adiponectin concentrations is [33, 34], which is known to be related to a different percentage of fat mass, usually higher in obese women compared to men. Low levels of adiponectin have been associated with the risk of developing type 2 diabetes or, less firmly, cardiovascular disease. The higher mean levels of adiponectin observed in our women may therefore be consistent with the generally more favourable anti-inflammatory and cardiovascular risk profiles with respect to men of similar age.

Differences in inflammatory parameters between women and men with metabolic syndrome have been previously reported. Sarbijani et al. [35] showed that IL-6 levels in men and malondialdehyde concentrations in women with metabolic syndrome were significantly higher than in control groups. Ahonen et al. [36] reported that differences in adiponectin in individuals with and without the metabolic syndrome were larger in women than men. Also, Ter Horst et al. [37] observed among subjects with the metabolic syndrome an excessive production of proinflammatory mediators, such as IL-6 and leptin, in men and a reduction in the anti-inflammatory adiponectin in women. They suggested that the role of inflammation in the adverse cardiometabolic consequences of obesity is different in women and men. Inflammation is linked to peripheral leptin resistance which increases leptin levels and other inflammatory mediators in males and to decreased adiponectin production in females.

In our study, we observed not only an inflammatory behaviour but also an active oxidative stress condition that may affect insulin resistance in a gender different way.

Sexual dimorphism was also present in myostatin concentrations with higher levels in females compared to males. This result may be also linked to gender differences in fat and muscle mass.

Myostatin, a member of the transforming growth factor-*β* (TGF*β*) family secreted by skeletal muscle, acts as a strong negative regulator of muscle development and size [13] and adipocyte differentiation and function [14]. In the experimental model, myostatin inhibition leads to an increase in muscle tissue, a decrease in fat component, and improved metabolism through increased metabolically active lean muscle mass. Conversely, the direct in vivo actions of myostatin on adipose tissue seem to be less relevant [15]. High muscle and serum myostatin concentrations have been documented in both mouse models and humans with obesity and found to be associated with systemic IR [16–18, 38]. Several lines of evidence suggest that myostatin is also an inducer of ROS in the cells and that ROS themselves are potent myostatin inducers. Sriram et al. observed that myostatin increases oxidative stress by producing ROS in skeletal muscle cells through tumor necrosis factor-*α* (TNF-*α*) signalling via NF-*κ*B and NADPH oxidase [39]. They also established that TNF-*α* and hydrogen peroxide are potent inducers of myostatin through NF-*κ*B activation. Interestingly, myostatin concentrations are correlated with plasma t-Hcy, ox-Hcy, r-Cys, t-GSH, and ox-GSH in our male group and are thus closely linked with the redox state alteration and increase in oxidative stress markers in these subjects.

However, we found no correlation with IR severity, in agreement with Toloza et al. [40] who observed no association between myostatin and any clinical IR indicator or direct IR index measure in both genders over a wide range of adiposity.

Pathways regulating myostatin serum concentrations in humans remain incompletely understood and require further investigation.

Our study has some limitations. The study series, although gender-balanced, was relatively small and restricted to middle-aged subjects, and thus, our findings may not be applied to the general population. We did not measure sex hormones to evaluate if their differential homeostasis may explain gender differences in inflammatory ad oxidative stress profiles. However, previous work demonstrated that no significant difference in sex hormone levels was associated with the metabolic syndrome in both women and men in the same age range [15]. Moreover, we did not compare our obese population with a lean control group. However, we considered a homogeneous sample with similar clinical characteristics able to identify the gender differences, which underlie a specific cardiometabolic risk profile.

## 5. Conclusion

In this study, we analysed the regulation of redox status and adipomyokine concentrations in obese individuals, and we showed that there is a sex-dependent association of severe metabolic alteration with some circulating markers of active oxidative stress and inflammation. Strict control of redox and inflammatory status, even setting different gender-specific nutritional targets, may be a useful tool to prevent obesity-related metabolic alterations and comorbidities in these subjects.

## Figures and Tables

**Table 1:** Anthropometric and clinical characteristics between gender.

	Female *N* = 31	Male *N* = 29	*P*
Age (years)	59 [57-64]	59 [55-63]	0.389
BMI	33 [32-35]	35 [32-37]	0.149
HOMA index	2.53 [1.80-5.11]	3.41 [2.37-5.53]	0.348
Glucose (mg/dL)	95 [90-110]	98 [95-107]	0.662
Glycated haemoglobin (mmol/mol)	37 [35-41]	36 [35-39]	0.334
Insulin (*μ*U/mL)	11.8 [7.8-18.8]	13.8 [10.1-21.1]	0.325
AST (U/L)	18 [16-22]	20 [18-26]	0.123
ALT (U/L)	18 [14-26]	21 [18-30]	0.087
HDL cholesterol (mg/dL)	56 [51-65]	44 [38-51]	<0.001
LDL cholesterol (mg/dL)	133 [113-150]	120 [108-144]	0.204
Triglycerides (mg/dL)	100 [81-121]	110 [84-145]	0.391
Visceral adipose index	3.39 [2.50-4.53]	3.50 [2.52-4.92]	0.830
Cardiovascular risk factors			
Smoking habit, *n* (%)	2 (7%)	7 (24%)	0.076
Hypertension, *n* (%)	13 (42%)	19 (66%)	0.077
Dyslipidemia, *n* (%)	12 (39%)	10 (35%)	0.793
Drugs and supplements			
Betablockers, *n* (%)	3 (10%)	4 (14%)	0.702
ACE-inhibitors or angiotensin receptor blockers, *n* (%)	8 (26%)	13 (45%)	0.176
Statins, *n* (%)	5 (16%)	2 (7%)	0.426
Vitamin D supplementation, *n* (%)	5 (16%)	0 (0%)	0.053

Data are expressed as median and interquartile range. HOMA: homeostasis model assessment.

**Table:** (a) Males (*N* = 29)

	Leptin (ng/mL)		Adiponectin (*μ*g/mL)		Myostatin (ng/mL)		Interleukin-6 (pg/mL)		Vitamin D (ng/mL)	
	Coef.	*P*	Coef.	*P*	Coef.	*P*	Coef.	*P*	Coef.	*P*
Plasma reduced homocysteine (*μ*mol/L)	-0.193	0.316	0.108	0.575	-0.112	0.570	-0.123	0.525	-0.062	0.750
Plasma total homocysteine (*μ*mol/L)	0.173	0.369	-0.100	0.608	0.423	0.025	0.007	0.972	-0.217	0.257
Plasma oxidized homocysteine (*μ*mol/L)	0.198	0.304	-0.098	0.613	0.428	0.023	0.014	0.943	-0.235	0.219
Plasma reduced cysteine (*μ*mol/L)	0.101	0.603	-0.079	0.683	0.423	0.025	0.295	0.121	0.169	0.381
Plasma total cysteine (*μ*mol/L)	0.261	0.172	-0.040	0.837	0.279	0.150	0.104	0.590	-0.169	0.381
Plasma oxidized cysteine (*μ*mol/L)	0.254	0.184	-0.061	0.755	0.262	0.179	0.088	0.649	-0.177	0.359
Plasma reduced glutathione (*μ*mol/L)	0.083	0.670	0.162	0.401	-0.029	0.885	-0.012	0.949	-0.139	0.473
Plasma total glutathione (*μ*mol/L)	0.109	0.575	0.056	0.772	0.474	0.011	0.051	0.795	-0.108	0.578
Plasma oxidized glutathione (*μ*mol/L)	0.156	0.418	-0.038	0.843	0.567	0.002	0.134	0.487	-0.074	0.703

**Table:** (b) Females (*N* = 31)

	Leptin (ng/mL)		Adiponectin (*μ*g/mL)		Myostatin (ng/mL)		Interleukin-6 (pg/mL)		Vitamin D (ng/mL)	
	Coef.	*P*	Coef.	*P*	Coef.	*P*	Coef.	*P*	Coef.	*P*
Plasma reduced homocysteine (*μ*mol/L)	0.091	0.625	0.087	0.642	0.052	0.781	0.061	0.745	-0.115	0.536
Plasma total homocysteine (*μ*mol/L)	0.074	0.691	0.169	0.365	0.058	0.756	-0.226	0.221	-0.092	0.623
Plasma oxidized homocysteine (*μ*mol/L)	0.055	0.769	0.158	0.395	0.080	0.668	-0.220	0.235	-0.090	0.630
Plasma reduced cysteine (*μ*mol/L)	-0.166	0.373	-0.071	0.703	0.057	0.759	-0.052	0.779	-0.111	0.552
Plasma total cysteine (*μ*mol/L)	0.014	0.940	0.108	0.563	0.046	0.804	-0.235	0.203	0.099	0.597
Plasma oxidized cysteine (*μ*mol/L)	0.018	0.924	0.124	0.506	0.032	0.862	-0.259	0.160	0.106	0.569
Plasma reduced glutathione (*μ*mol/L)	0.010	0.957	-0.041	0.827	0.103	0.580	-0.155	0.404	-0.076	0.686
Plasma total glutathione (*μ*mol/L)	0.079	0.671	0.064	0.734	0.026	0.889	-0.152	0.415	0.032	0.866
Plasma oxidized glutathione (*μ*mol/L)	0.036	0.848	0.206	0.267	-0.159	0.394	-0.120	0.519	-0.058	0.756

Coef: Pearson coefficient or Spearman rho coefficient correlation for parameters with normal or nonnormal distribution, respectively.

**Table 3:** Variables associated with male gender by logistic regression.

	Univariable			Multivariable		
	OR	95% CI	*P*	Adjusted OR	95% CI	*P*
Plasma reduced homocysteine (*μ*mol/L)	0.903	0.279-2.921	0.864			
Plasma total homocysteine (*μ*mol/L)	1.239	1.066-1.441	0.005	1.299	0.945-1.785	0.107
Plasma oxidized homocysteine (*μ*mol/L)	1.257	1.074-1.470	0.004			
Plasma reduced cysteine (*μ*mol/L)	0.864	0.725-1.029	0.101			
Plasma total cysteine (*μ*mol/L)	1.009	1.001-1.018	0.035	1.005	0.987-1.024	0.558
Plasma oxidized cysteine (*μ*mol/L)	1.010	1.001-1.019	0.027			
Plasma reduced glutathione (*μ*mol/L)	0.530	0.229-1.223	0.137			
Plasma total glutathione (*μ*mol/L)	1.421	0.916-2.204	0.117			
Plasma oxidized glutathione (*μ*mol/L)	2.034	1.178-3.514	0.011	6.506	1.152-36.736	0.034
Leptin (ng/mL)	0.851	0.780-0.928	<0.001	0.738	0.591-0.921	0.007
Adiponectin (*μ*g/mL)	0.860	0.773-0.957	0.006	0.739	0.573-0.952	0.019
Myostatin (ng/mL)	0.849	0.728-0.990	0.037	0.673	0.476-0.951	0.025
Interleukin-6 (pg/mL)	0.962	0.681-1.361	0.829			
Vitamin D (ng/mL)	0.929	0.870-0.993	0.029	0.897	0.780-1.031	0.125

Referent category = female. OR: odds ratio; CI: confidence interval.

**Table 4:** Univariable logistic regression analysis for severe IR (HOMA ≥ 3.8).

	HOMA < 3.8 (*N* = 35)	HOMA ≥ 3.8 (*N* = 25)	OR	95% CI	*P*
Male gender, *n* (%)	15 (43%)	14 (56%)	1.697	0.603-4.778	0.317
Plasma reduced homocysteine (*μ*mol/L)	0.27 [0.19-0.48]	0.20 [0.14-0.48]	0.276	0.035-2.187	0.223
Plasma total homocysteine (*μ*mol/L)	10.36 [7.86-14.54]	12.66 [9.18-15.06]	1.071	0.956-1.199	0.235
Plasma oxidized homocysteine (*μ*mol/L)	9.97 [7.82-14.35]	12.53 [9.05-14.71]	1.083	0.963-1.217	0.184
Plasma reduced cysteine (*μ*mol/L)	10.97 [9.10-13.65]	10.14 [8.84-13.55]	0.940	0.795-1.112	0.472
Plasma total cysteine (*μ*mol/L)	278 [251-321]	331 [274-391]	1.012	1.003-1.021	0.010
Plasma oxidized cysteine (*μ*mol/L)	265 [238-308]	320 [267-380]	1.012	1.003-1.021	0.009
Plasma reduced glutathione (*μ*mol/L)	1.30 [0.97-1.58]	0.89 [0.63-1.62]	0.598	0.259-1.380	0.228
Plasma total glutathione (*μ*mol/L)	4.33 [3.76-4.80]	4.63 [3.41-5.44]	1.176	0.777-1.779	0.443
Plasma oxidized glutathione (*μ*mol/L)	2.72 [2.46-3.50]	2.93 [2.46-4.47]	1.460	0.908-2.350	0.118
Leptin (ng/mL)	13.60 [5.30-23.70]	18.80 [12.65-27.20]	1.053	1.003-1.105	0.036
Adiponectin (*μ*g/mL)	14.73 [10.80-22.82]	12.69 [10.42-17.33]	0.903	0.819-0.995	0.039
Myostatin (ng/mL)	10.47 [8.27-12.84]	11.60 [10.23-15.31]	1.080	0.952-1.226	0.231
Interleukin-6 (pg/mL)	1.94 [1.21-2.38]	2.54 [1.95-3.38]	1.814	1.133-2.905	0.013
Vitamin D (ng/mL)	26 [21-30]	18 [14-32]	0.994	0.885-1.007	0.078

Referent category = HOMA < 3.8. HOMA: homeostasis model assessment; OR: odds ratio; CI: confidence interval.

**Table 5:** Multivariable logistic regression analysis for severe IR (HOMA ≥ 3.8).

	Adjusted OR	95% CI	*P*
Male gender	4.086	0.516-32.390	0.183
Plasma total cysteine (*μ*mol/L)	1.016	1.004-1.029	0.012
Leptin (ng/mL)	1.133	1.034-1.241	0.007
Adiponectin (*μ*g/mL)	0.872	0.751-1.012	0.072
Interleukin-6 (pg/mL)	2.174	1.088-4.344	0.028
Vitamin D	0.962	0.879-1.052	0.398

OR: odds ratio; CI: confidence interval.

## Data Availability

The data used to support the findings of this study are restricted by the Niguarda Ethics Committee (Comitato Etico Milano Area 3) in order to protect patient privacy. However, excel data are available from the corresponding author upon request.
